# Protocol of a randomised controlled trial of a novel brief psychological intervention for young people presenting to emergency departments in the UK with self-harm or suicidal ideation with recent self-harm: the SASH study (Supporting Adolescents with Self-Harm)

**DOI:** 10.1136/bmjopen-2025-101015

**Published:** 2025-09-14

**Authors:** Rose McCabe, Sally O’Keeffe, Dennis Ougrin, Stefan Priebe, Peter Martin, Yan Feng, Rachel Temple, Maria Long

**Affiliations:** 1 Department of Global, Public and Population Health and Policy, City St George’s University of London, London, UK; 2 Population Health Sciences Institute, Newcastle University, Newcastle upon Tyne, UK; 3 Wolfson Institute of Population Health, Queen Mary University of London, London, UK; 4 Department of Primary Care and Population Health, University College London, London, UK; 5 McPin Foundation, London, UK

**Keywords:** Suicide & self-harm, Clinical trials, Adolescents, Emergency Departments, Psychosocial Intervention

## Abstract

**Introduction:**

Self-harm is the strongest predictor of suicide in young people. Self-harm presentations to the emergency department (ED) are associated with repeat self-harm and suicide. Rapid follow-up contact after ED offers an opportunity to intervene before self-harm becomes an established coping strategy. Despite recent progress in self-harm treatment, currently, there are no evidence-based interventions to prevent future self-harm and suicide offered to young people after visits to the ED. Preliminary evidence suggests therapeutic assessment and rapid follow-up contacts may reduce self-harm and improve engagement in follow-up care. In this study, we assess the clinical and cost-effectiveness of a brief psychological intervention, supporting adolescents with self-harm (SASH), in addition to standard care in a randomised controlled trial, compared with standard care only. As per National Institute for Health and Care Excellence guidelines, standard care involves at least one follow-up by a mental health professional within 7 days of ED discharge.

**Methods and analysis:**

The SASH intervention comprises up to six follow-up contacts with a mental health professional delivered over approximately 2 months for young people and their carers using a solution-focused approach, shortly after presenting to the ED. Participants are aged 12–18, presenting to the ED with self-harm or suicidal ideation (with self-harm in the past month), with capacity to consent. We aim to recruit 144 young people into the trial who will be randomised on a 1:1 basis to the SASH intervention or treatment as usual. Participants are assessed postintervention/standard care and at 6-month follow-up after randomisation. Self-reported self-harm is assessed via text message survey every 2 weeks during the 6-month follow-up period. The primary outcome is self-reported episodes of self-harm in the past month assessed at 6 months by summing three behavioural domains of the self-injurious thoughts and behaviours interview. We hypothesise that the therapeutic relationship with the mental health practitioner will mediate this relationship. Secondary outcomes include symptoms of depression and anxiety, frequency of reattendance at ED, death by suicide, school attendance, well-being and additional domains of self-harm-related behaviour and thoughts in the past month. The trial will also consider service use, costs to carer and carer health-related quality of life to evaluate the costs and cost-effectiveness of the intervention.

**Ethics and dissemination:**

London-Riverside Nation Health Service REC (22/LO/0400) provided a favourable ethical opinion. Findings will be disseminated through social media, a website, scientific papers, conferences and reports, in collaboration with our Young Person’s Lived Experience Advisory Group.

**Trial registration number:**

ISRCTN81846131.

**Protocol version:**

13.0, 30.06.2025.

**STRENGTHS AND LIMITATIONS OF THIS STUDY**

- Broad inclusion criteria maximise the generalisability of the findings to other settings, which offer care to young people who self-harm.
- Trained practitioners deliver the intervention in the intervention arm, and a different group of practitioners deliver standard care received in the Treatment As Usual (TAU) arm, mitigating contamination between the two arms.
- The intervention is designed to be delivered by practitioners from different professional groups.
- Standard care differs across different sites, introducing heterogeneity in the TAU group.

## Background

 Suicide is the second-leading cause of death in young people globally.^1^ Death by suicide is associated with previous self-harm, defined as ‘intentional self-poisoning or injury irrespective of the apparent purpose of the act.’^2^ A national study found that, in England, over 50% of those under 20 who die by suicide have a history of self-harm.^3^ A meta-analysis of community samples 1990–2015 across 41 countries found that the overall lifetime prevalence of self-harm in 12–18 years old is 16.9%.^4^

A meta-analysis, of which approximately 40% of the participants are under the age of 24, found that presenting to the emergency department (ED) with non-fatal self-harm is associated with a nearly 50-fold increased risk of suicide across all ages.^5^ In England, the National Institute for Health and Care Excellence (NICE) recommends a psychosocial assessment by specialist mental health practitioners in the ED for young people who present with self-harm, along with a follow-up within 7 days of discharge.^2^ Practitioners conduct psychosocial assessments to assess current mental health, risks, needs and agree a management plan. Follow-up within 7 days involves appropriate aftercare, including risk assessment, a mental state exam and any onward referrals, including referrals to community Children and Adolescent Mental Health Services (CAMHS) teams. Meta-synthesis of the limited literature on young people’s experiences of mental healthcare in the ED suggests that many young people are not satisfied with care in the ED.^6^ Young people report their distress being dismissed^7^ and receiving formulaic assessments instead of compassionate care.^8^ This impacts on engagement in follow-up care, deters help seeking and leads to a vicious cycle of repeat ED attendances and escalating suicidality.^7 9^ Where follow-up care is received, it is often perceived as unhelpful, focusing on generic advice, such as calling a helpline or crisis number, rather than personalised solutions.^8^ This aligns with other research where young people emphasised the need for compassionate, personalised care to break the cycle of self-harm.^10^

Reducing self-harm is a major public health concern, and despite recent progress in treatments for self-harm,^11 12^ there are few interventions specifically designed for young people,^13 14^ especially following ED attendance. Designed for young people presenting with self-harm and derived from solution-focused and systemic traditions, Therapeutic Assessment (TA) is a promising approach, which may help young people break the cycle of self-harm.^15^ TA is a collaboratively designed formulation, showing the links among the young person’s thought processes and core pain, their self-harm and its short-term benefits and long-term consequences and how this feeds cyclically into the young person's core pain.^15^ It also addresses where the cycle of self-harm can be broken. Safety plans^16^ focus on warning signs, internal and external coping strategies, informal and formal support and restricting access to means of self-harm. Solution-focused sessions focus on what the young person wants to be different in their future, when the problem is not happening, their strengths, what is already working and how to make small steps towards what is wanted in their future.

Evidence from a non-randomised study found that young people who received TA were less likely to harm themselves.^17^ A randomised controlled trial of TA found that, compared with Treatment As Usual (TAU) (standard psychosocial assessment), TA increased engagement in follow-up care,^18^ which is a key mechanism for reducing future self-harm. This is consistent with a systematic review of the evidence in adults, which concluded that the components of effective interventions following ED presentations for self-harm are therapeutic assessment, safety planning and rapid follow-ups.^19^ Building on this evidence, the Supporting Adolescents with Self-Harm (SASH) intervention was developed for 12–18-year old to be delivered soon after ED attendance. The age range reflects the ages of young people seen by participating services, which is also in line with other trials of TA,^18^ and takes into account a child’s developmental stage in terms of their ability to engage in a psychological intervention. The intervention involves up to six sessions and includes TA of self-harm,^15^ enhanced safety planning^16^ and up to five solution-focused sessions over 8 weeks.

## Objectives

The objectives of the SASH study are as follows.

1. To assess whether the SASH intervention, in addition to standard care, reduces self-harm in young people who present to the ED with self-harm or suicidal ideation (with self-harm in the past month), compared with a TAU group who receive standard National Health Service (NHS) care only. To assess whether the SASH intervention improves secondary outcomes, including suicidal ideation, symptoms of depression and anxiety, school attendance, repeat ED attendance for self-harm or suicidal ideation, suicide and well-being.
2. To explore how the intervention is experienced by participants, including minority ethnic, non-heterosexual and gender-diverse young people, and how it should be adapted appropriately for diversity among young people (eg, ethnicity, sexuality and gender).
3. To assess the costs and cost-effectiveness of the intervention.

## Methods and analysis

Reporting of methods is in line with the Standard Protocol Items: Recommendations for Interventional Trials checklist in the online supplemental material.

### Study setting and sites

The trial is a multicentre study sponsored by City St George’s, University of London. The trial will recruit young people who have presented to one of the nine different EDs in London, UK. Young people presenting to an ED are seen by practitioners from six CAMHS crisis/urgent care teams. The six CAMHS crisis/urgent care teams are a part of three NHS Trusts or organisations: East London NHS Foundation Trust, Central and North West London Foundation NHS Trust and West London NHS Trust. We aim to recruit within 48–72 hours of discharge from the ED.

### Eligibility criteria

#### Young people participants

##### Inclusion criteria

- 12-18 years old
- Presenting in crisis to the ED with self-harm, according to the UK NICE definition, as ‘intentional self-poisoning or injury irrespective of the apparent purpose of the act’,^2^ or suicidal ideation with recent self-harm defined as within 1 month of ED presentation

##### Exclusion criteria

- Possible learning disability, judged by a clinician, due to potential engagement difficulties with consent and study procedures.
- Need for more intensive treatment than the intervention offers, eg, inpatient treatment (tier 4) or intensive/outreach care in the community (tier 3.5).
- Current psychotic episode.
- Registered with a General Practitioner (GP) outside of the mental health NHS Trust catchment area.
- Receiving individual one-to-one psychological therapy for more than 1 hour per week.
- Interpreter required to complete research procedures.

### Practitioner participants

NHS practitioners working or allied with CAMHS crisis/urgent care/community teams delivering follow-up care to young people after presenting to ED with self-harm (eg, mental health nurses, social workers, assistant psychologists and clinical associate psychologists).

### Parent/carer participants

Parents/carers with parental responsibility of young people participants under the age of 16. Parents/carers of young people over the age of 16 are also invited to participate where preferred by the YP.

### Consent

University or NHS researchers with training in Good Clinical Practice and the study procedures will obtain informed consent. A guardian with parental responsibility provides consent for young people under the age of 16. Young people participants under the age of 16 indicate their willingness to participate by providing assent after an age-appropriate explanation of the study from a researcher. Participants aged 16 or over provide informed consent for themselves. Researchers receive assent/consent either remotely or in person after full discussion of the study. See the online supplemental material for a participant consent form.

### Trial design

**Figure 1:**
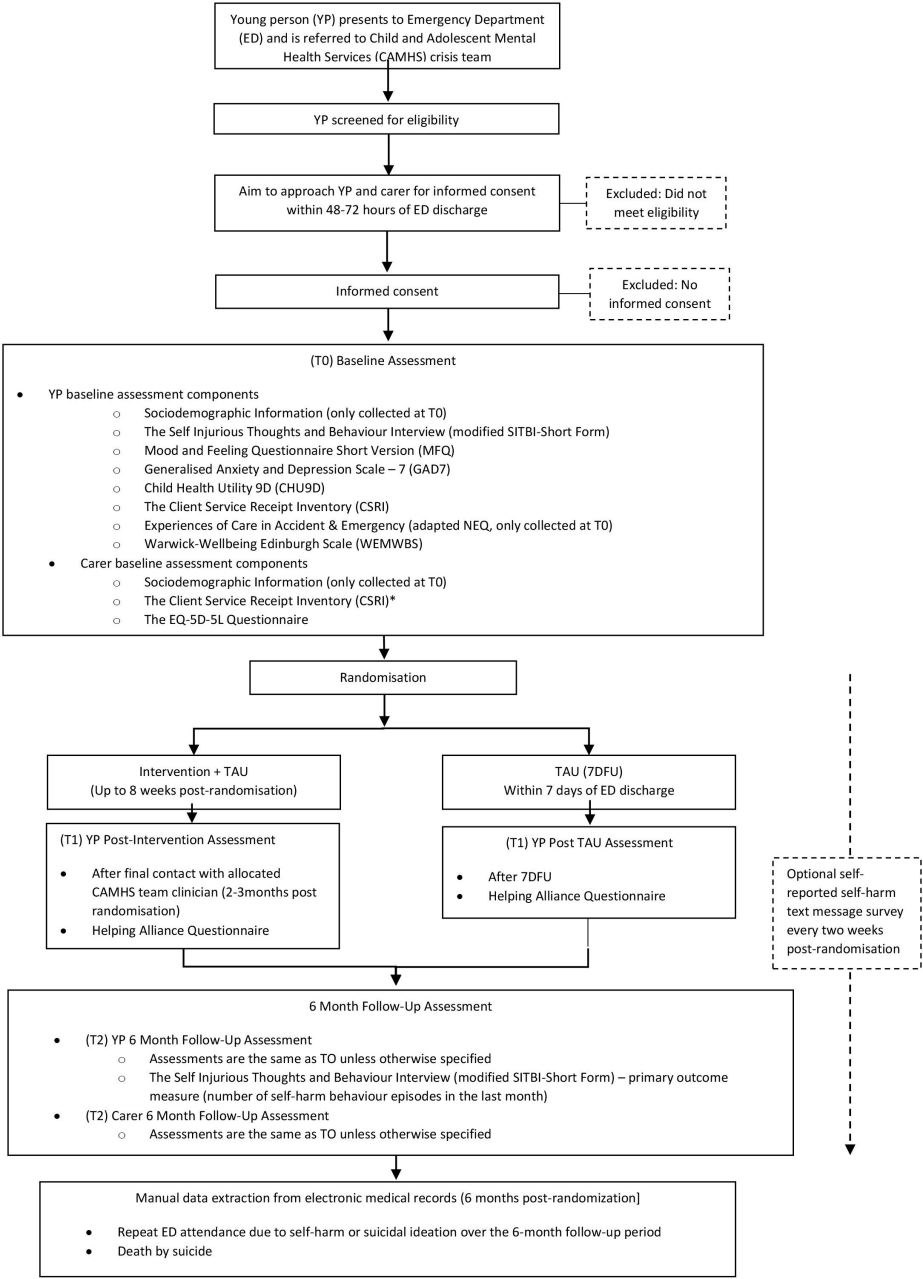
Trial design flowchart. TAU, treatment as usual.

See figure 1 for the trial design flowchart. The trial consists of a parallel group individually randomised trial with concealed allocation. Randomisation is conducted using permuted blocks and is stratified by CAMHS crisis team and whether the young person has presented to the ED more than one time (binary variable). Randomisation is conducted with 1:1 allocation through a secure internet-based system (REDCap). The randomisation sequence is generated by an independent, external statistician. Participants and clinicians are informed of allocation. The follow-up period is 6 months, with two follow-up assessments; the first is conducted postintervention (the intervention lasts around 8 weeks) or after the 7-day follow-up for young people allocated to TAU; the second and final assessment is conducted 6 months postrandomisation.

#### Blinding

Outcome assessors are blinded where possible, which is achieved with support from blinded researchers who are not exposed to information on the outcome of randomisation or the monitoring of the intervention. Blinded research staff record if they suspect allocation after conducting the follow-up assessment. The statistician will be blinded to treatment allocation until the findings of the primary outcome analysis are confirmed and have been approved by the project team. SASH trial health economists will remain blind to participant allocation until the health economics analysis plan (HEAP) has been signed off and the trial database finalised and locked for analysis.

### Intervention development

The SASH intervention was developed in consultation with clinicians, academics and in collaboration with the SASH Young Person's Advisory Panel (YPAG). While the intervention consists of three distinct therapeutic elements, solution-focused practice underpins the whole intervention. Together, young people and practitioners jointly construct the young person's cycle of self-harm.^15^ A safety planning intervention based on the Stanley and Brown’s model^16^ and drawing on the principles of behaviour change was adapted for use with young people and codeveloped with the YPAG. Rapid follow-up contacts took a solution-focused approach and involved both young people and their parents/carers. Parents/carers can also receive individual sessions. The intervention is designed to be generic and delivered by CAMHS clinicians from any professional background. Through discussion with members of the project management group with expertise in psychiatry, psychotherapy, trials, lived experience and the YPAG, the intervention was refined and manualised. For example, after discussion of the safety plan with the YPAG, a section that prompts the reflection of the young person's qualities, needs, preferred activities and barriers to well-being was added to foster rapport and a personalised approach.

#### Training and supervision of practitioners

A broad range of CAMHS NHS practitioners are trained to deliver the intervention (bands 4–7), as specified by local service requirements for the seniority and experience level of clinicians who can deliver TAU. CAMHS clinicians are trained online or face-to-face across 1.5–2 days in a group format, though one-to-one sessions can be provided if necessary. Training is provided by the research team. Practitioners learn about the intervention through taught content, training videos of TA, safety planning, solution-focused sessions and role plays.

In each team, there are designated SASH-trained practitioners and TAU practitioners. SASH-trained practitioners are instructed in how to mitigate the contamination of their practice by not sharing SASH intervention techniques with TAU colleagues. A SASH intervention fidelity scale is applied to the medical records (entered by practitioners) for a random sample of intervention cases. The same scale is applied to a random sample of TAU cases to monitor contamination between the two arms. Supervision is offered in a group format on a weekly basis with senior members of the SASH research team.

### Intervention arm

The SASH intervention constitutes up to six sessions with young people and their carers: we aim to deliver the first session within 7 days of ED attendance. Subsequent sessions are delivered at approximately 2-week intervals. Where eligible young people are still in crisis or are uncontactable after ED, the intervention can be delivered after the 7-day follow-up (TAU). Sessions are primarily delivered face-to-face, though remote delivery is also possible depending on NHS Trust policies; intervention delivery is designed to be flexible to improve session attendance in terms of format, delivery and session intervals. The first session includes TA and enhanced safety planning. TA involves the practitioner and young person drawing a diagram of the self-harm cycle, with three elements: identification of core pain, maladaptive procedures and means to break the cycle. Enhanced safety planning involves coconstructing an individualised safety plan using the young person's words. This consists of details about the young person's qualities, preferred activities, needs, barriers to well-being, warning signs of self-harm and means of managing thoughts of self-harm, including individual actions (distractions and changing environment) and support networks (contacting trusted others and professionals).

The subsequent sessions use a solution-focused approach, which explores what the young person wants to be different in their future, when the problem is not happening, their strengths, what is already working and how to make small steps towards what is wanted in their future. Parent/carer involvement may include joint sessions with the young person or up to two individual sessions where clinicians take a solution-focused approach or offer psychoeducation and/or emotional support to the carer. TAU (standard care) is received concurrently. In response to changes in risk or worsening of presentation, clinicians can refer young people on for more support, or for specialist assessment. Fidelity to the intervention will be ensured through use of the manual, intervention handouts and regular peer supervision. Fidelity is monitored via regular medical notes review and practitioner-reported data.

### TAU arm

Young people in the TAU arm receive standard care following ED attendance for self-harm, namely a follow-up within 7 days of ED attendance, which is based on NICE guidelines. Follow-up includes the ongoing assessment of risk, need and onward referrals where necessary. CAMHS care is heterogeneous, with some CAMHS teams offering more than one follow-up after ED. These are captured as a part of routine trial reporting.

### Recruitment

We aim to randomise 144 young people who present to the ED, between May 2023 and April 2025. Young people and their carers will be invited to participate either by a practitioner at the point of attending the ED or shortly after discharge by a local NHS researcher. We aim to randomise young people within 72 hours of discharge from the ED. Where young people/carers are uncontactable, randomisation will occur a maximum of 1 month following discharge from ED.

### Outcomes

Outcome measures are administered using a case report form (CRF) by trained researchers. Some secondary outcome data are collected from medical records, as specified below. Data collection timelines are shown in table 1.

**Table 1:** Participant timeline

Assessment	Baseline	Conducted after completion of intervention or 7-day follow-up	Follow-up (6 months)	Follow-up (every 2 weeks)
Young people participants				
Modified short form SITBI	X		X	
MFQ	X		X	
GAD-7	X		X	
CHU9D	X		X	
WEMWBS	X		X	
CSRI	X		X	
Self-harm text message survey				X
HAQ		X		
Semi-structured interviews exploring experiences of the intervention		X		
Carer participants				
CSRI	X		X	
EQ-5D	X		X	
Semistructured interviews exploring experiences of the intervention		X		
Practitioner participants				
Demographics	X			
Professional background	X			
Semistructured interviews		X		

CSRI, administered to carer participants if YP <16 years and YP participants if YP >16 and their carer is not a participant in the study.

CHU9D, child health utility 9-D; CSRI, client service receipt inventory; GAD-7, general anxiety disorder-7; HAQ, helping alliance questionnaire; MFQ, mood and feelings questionnaire; SITBI, self-injurious thoughts and behaviours interview; WEMWBS, Warwick–Edinburgh mental well-being scale; YP, young person.

#### Primary outcome

The primary outcome is the number of self-reported episodes of self-harm within the past month, assessed 6-months postrandomisation. This is a count outcome based on summing the number of episodes of self-harm reported from a single item (‘How many times in the past month?’) across the three behavioural domains (suicide attempt, self-harmed without intent to end life and self-harm with ambiguous intent) from a modified version of the short form of the self-injurious thoughts and behaviours interview (SITBI).^20^ The term ‘Suicide Gesture’ has been criticised for being dismissive and for potentially minimising the seriousness of suicidal behaviour. Hence, there is agreement that it should be adapted^21^: we used ‘self-harm with ambiguous intent’. Young people are asked to report on the number of times in the past month they have attempted suicide, self-harmed without intending to end their life or self-harmed where the intent behind the act was ambiguous. These three domains encompass all possible episodes of self-harm in line with UK NICE definition.^2^ The SITBI has been widely used in young people who harm themselves and has strong validity and reliability.^20^

#### Mediator of effect on primary outcome

Quality of the therapeutic relationship, self-rated by young people participants and measured by the helping alliance questionnaire.^22^

##### Secondary outcomes

- Depressive symptoms, assessed with the short version of the mood and feelings questionnaire.^23 24^
- Anxiety symptoms assessed by the general anxiety disorder-7.^25^
- Repeat ED attendance due to self-harm or suicidal ideation over the 6-month follow-up period, identified in medical records.
- Death by suicide, that is, cause of death is intentional self-harm or undetermined intent, identified in NHS records.
- School attendance, obtained from the client service receipt inventory (CSRI),^26^ is a secondary outcome because of the relationship between self-harm and school absenteeism.^27^
- Mental well-being assessed by the Warwick–Edinburgh mental well-being scale.^28^
- Three separate domains of self-harm behaviour assessed by the modified SITBI short form: number of reported episodes of self-harm with suicidal intent, self-harm without suicidal intent and self-harm where intent is ambiguous in the past month.^20^
- Three separate domains of self-harm-related suicidal ideation assessed by the SITBI short form: number of reported episodes of suicidal ideation, suicide plans and thoughts of non-suicidal self-injury in the past month.^20^
- Dichotomised self-harm (any self-harm in the past month) assessed by the modified SITBI short form.^20^
- Number of young person reported self-harm episodes obtained every 2 weeks for the duration of 6-month follow-up via text message survey where provided.
- Parent/carer self-reported health-related quality of life assessed by the EQ-5D-5L.^29^
- Young people’s self-reported health-related quality of life data collected using Child Health Utility 9-D (CHU9D).^30^

##### Additional variables

- Experiences of care in the ED assessed by a version of the Negative Effects Questionnaire^31^ developed for a similar project in adults, the ASSURED trial.^32^
- Parental/carer involvement in care received as a part of the intervention or TAU (binary variable), captured as a part of a bespoke form designed specifically for the SASH trial.

##### Data that will be extracted from records

- Co-occurring mental disorders.
- Young people and carer participants' sociodemographic and clinical baseline data from medical records will be complemented with interviews.

##### Resource use will also be assessed

- Resources to train SASH practitioners and resources involved in delivering SASH and TAU will be collected using two health economics inventory forms designed for the SASH trial.
- Health and social care service use and school support services use by young people will be assessed using the CSRI.^26^
- Productivity loss and family resource use due to the mental health difficulties of young people will be assessed using the CSRI.^26^

##### Practitioner-reported data

- Demographic data.
- Participant attendance at follow-up sessions.

Outcome data will be collected from all participants. Retention in the trial will be maximised by conducting research assessments out of hours to accommodate young people and carer schedules and commitments. Measures will be completed flexibly via telephone, video conference, email or via text message if appropriate. Retention will also be enhanced by sending regular ‘staying in touch’ texts with study updates and thank you cards.

### Patient and public involvement

A YPAG was formed by the McPin Foundation. This consisted of 11 young people with lived experience of self-harm, suicidality and/or experience of ED care. The group was coordinated and chaired by two young adults with lived experience of mental health issues. In the development phase of the project, the team met with the YPAG every 3 months to advise on study design and to codevelop the SASH approach. For example, valuable feedback from the YPAG resulted in removing a previous exclusion criterion, excluding young people with autism from participating. Additional input included advice on progress, reviewing study materials and dissemination, co-creation of training videos and piloting measures. The YPAG plays a key role in advising on recruitment and retention, ethics and shaping operational procedures and dissemination. Members of the YPAG will be involved in the interpretation of all findings, in particular, the qualitative interviews.

### Independent committees

The trial has an independent project steering committee (PSC) and a data and ethics monitoring committee (DMEC). Both include a clinical academic, statistician, expert by lived experience and trialist. The DMEC ensures the safety, rights and well-being of participants by monitoring study progress, data and intervention completion rates, safety reporting and advising on ethical issues, making any relevant recommendations to the PSC.

### Data management

Personal information is stored at NHS Site or with the Sponsor, with hard copies stored in locked files, and electronic versions stored on encrypted servers, accessed by the study team only. Data will be entered by researchers into a secure bespoke RedCAP database stored on Sponsor servers and designed specifically for the trial with appropriate range checks for values. Adverse events are recorded in logs and their relatedness to the intervention is determined by site Principal Investigtors (PIs) who are all senior clinicians. Data monitoring is undertaken by the research team in collaboration with the Sponsor and recruiting sites.

### Analyses

#### Statistical analyses

The primary outcome is the number of episodes of self-harm in the last month measured 6 months after randomisation. This is a count outcome. Preliminary data^17^ suggest that the number of self-harm episodes is overdispersed in this population. A mixed-effects negative binomial regression model will estimate the effect of the intervention on the number of self-harm episodes, compared with TAU only. The model will adjust for number of self-harm episodes in the month prior to treatment and clustering of participants within crisis teams. The evidence for the effectiveness of the intervention will be assessed by a two-sided z-test of the log rate ratio, at *α*=0.05. Our primary analysis will be intention-to-treat. A per-protocol analysis will also be carried out. Sensitivity analyses will evaluate the robustness of conclusions to various statistical assumptions by fitting an additional random intercept for practitioners in the primary analysis model and by using different types of count model instead of negative binomial regression.

Exploratory analyses will be conducted on the fortnightly longitudinal measures of self-harm via text message survey. If sufficient data from the text message surveys are available, a longitudinal model for change in self-harm will be developed to investigate the trajectory of change and whether this varies between different subpopulations, especially minority ethnic, non-heterosexual and gender-diverse groups.

Secondary outcomes will be investigated using generalised linear mixed-effects models, accounting for clustering within crisis teams via random intercepts and using error distributions as appropriate depending on the measurement level and distribution of each secondary outcome.

Exact details of all analyses will be described in a statistical analysis plan, which will be finalised and signed off before the master database is locked on completion of data collection.

#### Sample size calculation

The measure of effect size is the rate ratio: the relative rate of the number of self-harm episodes post-treatment in the intervention group compared with the TAU group. A 33% reduction in self-harm episodes, relative to TAU, would constitute a clinically meaningful effect. This corresponds to a rate ratio of 0.67 and is at the ‘small effect’ end of the range of rate ratios observed in similar studies in adults.^1933^,^35^

Count outcomes used in trials are often overdispersed,^36^ and the amount of overdispersion (ie, the variance) has a large effect on statistical power.^37^ There is little published information about the variance of self-harm episodes per month in our target population. English *et al*^17^ present statistics on the number of self-harm episodes 6 months after TA based on n=14. They observed a mean of 1.1 with an SD of 1.6, indicating considerable overdispersion.

In the sample size calculation, we assumed that the pretreatment number of self-harm episodes would follow a negative binomial distribution. Standard measures to adjust the sample size for clustering within practitioners (within-practitioner intraclass correlation coefficients (ICC)) and adjustment for self-harm at baseline (within-participant correlation) apply to linear models and cannot strictly be applied to a count regression model. Given the sparsity of information about the true distribution of our outcome, we decided to use reasonable approximations in our power calculations. Thus, we used sample size formula for negative binomial models published in Zhu and Lakkis article and considered, as a rough approximation, sample size adjustment based on classical design effect and baseline adjustment formulae. Based on a previous study of self-harm in adults, where the within-practitioner ICC was<0.01, we estimated the within-practitioner correlation as 0.01 (In Rozental *et al*’s study,^31^ a smaller ICC would result in higher statistical power). With 44 practitioners participating, the design effect is deff=1 + 0.01 × (44 – 1) = 1.43. We estimated the within-participant correlation to be 0.5, a conservative estimate (Tamborrino^36^ found a median of 0.59 from 123 studies—a higher within-participant correlation would mean higher power). This implies a downward adjustment of the sample size by a factor of 1–0.552=0.7. Thus, the loss of power due to the design effect and the gain of power due to baseline adjustment are likely to approximately cancel each other out (since 1.43×0.7 ≈ 1). We further assumed no within-site correlation, which is again conservative (a non-zero correlation within study sites would mean higher power). With these parameters and using Gysin-Maillart *et al*^35^ formulae, an overall sample of 122 young people (61 per group) would yield 80% power if the overdispersion is at most 0.28. Power would be higher if the overdispersion was smaller. Attrition was estimated at 15%, estimated based on 5%–25% attrition rate found in similar trials in young people depending on the duration of follow-up.^38 39^ Thus, our target sample size is 144. Given the estimated 44 practitioners overall, we would need to recruit 3–4 participants per practitioner.

We performed a sensitivity check of our power analysis using a simulation approach with the simr package^40^ in R.^41^ We simulated data with the required properties, translating our estimates of within-participant correlation into a log rate ratio and within-practitioner correlation into a random intercept variance, and fitted an overdispersed Poisson regression model. Using 40 practitioners, total n=120 and 1000 simulated model runs, we found that overdispersion may need to be 0.15 or smaller for power to be reasonably certain to be at least 80% (estimated power: 83.7%; 95 % CI 81.3% to 85.9%).

#### Qualitative process evaluation

A qualitative process evaluation will be conducted using semistructured interviews with a purposively selected group of young people, parents/carers, trained practitioners and other staff in participating teams. Interviews will be audio recorded, transcribed and analysed using thematic analysis.^42 43^ Topic guides have been developed with support from the YPAG.

We will interview approximately 20 young people who received the SASH intervention to explore their experiences of the intervention, explore changes to managing self-harm and their well-being overall. Purposive sampling will be used to select young people with differential characteristics in terms of gender, ethnicity, whether or not they completed the intervention and whether the index ED visit was the first ED attendance or not. Interviews will be conducted soon after intervention completion to aid recall. Ten parents/carers will also be interviewed. These will be sampled based on whether or not they directly participated in the intervention. We will interview approximately 20 practitioners and other staff in participating teams, who will be purposively sampled according to their professional background and years of experience. We will also interview up to 20 young people and parents/carers in the TAU arm to explore their experiences of standard care. Purposive sampling will be used to select based on the demographic characteristics, such as sex, age, ethnicity and sexual orientation, and characteristics of the care received after ED presentation. Parents/carers will also be sampled based on their relationship with the young person.

#### Fidelity to the intervention

Intervention fidelity will be assessed through review of medical records by the research team using a fidelity scale developed and piloted by the research team.

#### Economic evaluation

The economic evaluation aims to assess the cost-effectiveness of the SASH intervention in comparison with TAU. Our evaluation will follow the intention-to-treat principle. The analysis will adopt the NHS and personal social services perspective.^44^ The time horizon of the evaluation will start from baseline until the end of the 6-months follow-up. No discount will be required for costs and outcomes data analysis.

The resource use will include: (1) resource use relating to training SASH practitioners who deliver the SASH interventions; (2) health and social care services and school support services use by young people and (3) productivity loss for parents/carers and family resource use due to the mental health difficulties of young people. We will collect resource use data from practitioners training and intervention delivery during the SASH trial using two health economics inventory forms that are designed for the trial. We will invite carers to fill in an adapted CSRI at baseline and 6-months follow-up for young people’ use of health and social care services and school support services. Furthermore, carers will complete the adapted CSRI for the impacts of the YP’s mental health difficulties on the family, including carers being absent from work and various types of resource use due to childcare. Costs of each resource use item will be calculated as a product of the quantity of resource use and its corresponding unit cost. Most unit costs data will be taken from publicly available data sources. We will collect the salary data for practitioners and carers using one of the health economics inventory forms and the adapted CSRI, respectively. Cost items will be summed together and presented at the young person level. When it is not possible to collect CSRI data from carers, we will ask young people participants to self-report relevant sections of the CSRI.

The primary outcome measure in economic evaluation will be quality-adjusted life years (QALYs), converting from CHU9D utility scores.^30^ We will also collect carers’ health status data using the EQ-5D-5L instrument.^29^ Both CHU9D and EQ-5D-5L data will be collected at baseline and 6-months follow-up using the CRF. In the primary economic evaluation, we will conduct cost utility analysis to estimate the incremental costs for the SASH intervention in comparison with TAU for each additional QALY gained. In the sensitivity analysis, we will explore the impact of the missing data assumption. A scenario analysis will be conducted to include carers’ outcomes (measured by the EQ-5D-5L instrument) and costs to family (measured by carers’ productivity loss and family resource use due to the mental health difficulties of young people) in a cost utility analysis. Finally, we will report the evaluation result from a cost-effectiveness analysis by applying the number of self-harm episodes in the last month measured at 6 months after randomisation as the outcome measure (instead of QALYs converted from the CHU9D utility scores).

Details of the economic evaluation will be described in the HEAP.

## Ethics and dissemination

The study was reviewed and received a favourable opinion by the London-Riverside NHS Ethics Committee (22/LO/0400). Any study amendments are overseen by the Sponsor and communicated by the research team to the relevant stakeholders. Once primary analysis is complete, the dataset may be made available on request. Dissemination of study activities to the public will take place via the study website (https://sashstudy.co.uk/) and X channel (@SASHStudyCity). We will develop an accessible dissemination plan with the YPAG for young people audiences. Results will be accessible through open-access publications in peer-reviewed scientific journals, presentations at national and international conferences and through existing networks in NHS England, Integrated Care Boards, NHS Trusts and third sector organisations in suicide prevention and lay reports in collaboration with the YPAG.

### Data statement

The final dataset will be accessed by the study team. After primary analysis, the datasets generated and/or analysed during the current study will be available on request—where relevant consent is in place—from Prof Rose McCabe (rose.mccabe@citystgeorges.ac.uk). Individual-level patient data will not be made publicly available due to data privacy/General Data Protection Regulation (GDPR). Additional access to the final study dataset will be considered with an appropriate data-sharing agreement in place.

## Supplementary material

10.1136/bmjopen-2025-101015 online supplemental file 1

10.1136/bmjopen-2025-101015 online supplemental file 2
