# Supplementary material for: Protocol of a randomised controlled trial of a novel brief psychological intervention for young people presenting to emergency departments in the UK with self-harm or suicidal ideation with recent self-harm: the SASH study (Supporting Adolescents with Self-Harm)
Source: BMJ Open. 2025 Sep 14;15(9):e101015. doi: 10.1136/bmjopen-2025-101015 (PMC12434769; doi:10.1136/bmjopen-2025-101015)
Supplement: online supplemental file 2 [file bmjopen-15-9-s002.docx]

**YOUNG PERSON REMOTE CONSENT FORM (AGES 16-19, Part One)**

**SASH Study: Supporting Adolescents with Self-Harm**

*Researcher guidance: Please, complete this form if you are required to receive consent from a patient remotely, i.e. over a telephone conversation. Read each point carefully and allow time for patients to ask questions. Ensure that you have correctly entered Patient ID in both pages and add* ***your*** *initials in each box accordingly. At the end of telephone conversation participants must be sent the complete remote consent form to their emails or post. Ensure that you explain that participants have 3 days to raise any concerns or express their disagreement.*

*Please* ***initial*** *the relevant* *box*

|  | Yes | No |
| --- | --- | --- |
| 1. I have read and understood the full information sheet dated XX/XX/XXXX Version X.0 including how my data will be retained and used. I have had a chance to ask questions and they have been answered to my satisfaction. |  |  |
| 1. It is my choice to take part and I am free to withdraw at any time without giving a reason. |  |  |
| 1. I agree to be contacted by a researcher about this study. |  |  |
| 1. The researchers may access my medical records for sociodemographic and healthcare information about me. |  |  |
| 1. My GP will be informed about my participation in this study. |  |  |
| 1. All my information will be kept confidential and secure. |  |  |
| 1. I agree to take part. |  |  |

**PLEASE CONTINUE ON NEXT PAGE**

| *The following items are optional. Please* ***initial*** *box accordingly* | | |
| --- | --- | --- |
|  | Yes | No |
| 1. I would like to receive a copy of the final research report. |  |  |
| 1. I understand that other researchers may use information collected about me where I cannot be recognised in future research, e.g., questionnaires. |  |  |
| 1. I understand that other researchers may use information collected about me where I can be recognised in future research, e.g., audio and video recordings. |  |  |
| 1. I agree to be invited to participate in future research projects. I may opt out of receiving these invitations at any time. |  |  |
| 1. I agree to be interviewed about my experiences of the study, which will be audio-recorded. The recording will be written out, names and locations that can identify me will be removed, and sections will be used in publications and presentations. |  |  |
| 1. I agree to be recorded during the meetings with the mental health professional, for training and presentation purposes. (*If yes, please complete the Part Two form)* |  |  |

I (Name of Researcher) _________________________, (Research Role) _________________

of the SASH Trial research team can confirm that on the (date) ________________ obtained consent from (Name of Participant) ______________________________ remotely over the phone. Participant would like to receive a copy of this consent form via email/post (please circle)

____________________________________ __________________ __________________________

Name of the Researcher Date Signature
