# Supplementary material for: Protocol of a randomised controlled trial of a novel brief psychological intervention for young people presenting to emergency departments in the UK with self-harm or suicidal ideation with recent self-harm: the SASH study (Supporting Adolescents with Self-Harm)
Source: BMJ Open. 2025 Sep 14;15(9):e101015. doi: 10.1136/bmjopen-2025-101015 (PMC12434769; doi:10.1136/bmjopen-2025-101015)
Supplement: Supplementary file 3 [file bmjopen-2025-101015.reviewer_comments.pdf]

PEER REVIEW HISTORY

BMJ Open publishes all reviews undertaken for accepted manuscripts. Reviewers are asked to complete a checklist review form and are provided with free text boxes to elaborate on their assessment. These free text comments are reproduced below.

ARTICLE DETAILS

Title (Provisional)

Protocol of a randomised controlled trial of a novel brief psychological intervention for young people presenting to Emergency Departments in the UK with self-harm or suicidal ideation with recent self-harm: the SASH study (Supporting Adolescents with Self-Harm)

Authors

McCabe, Rose; O'Keeffe, Sally; Ougrin, Dennis; Priebe, Stefan; Martin, Peter; Feng, Yan PhD; Temple, Rachel; Long, Maria

---

VERSION 1 - REVIEW

**Reviewer**	**1**
**Name**	**Cwik, Mary**
**Affiliation**	**Johns Hopkins University**
**Date**	**01-May-2025**
**COI**	**None**

Very clear description of the methods for this important study. A few minor comments for the authors.

1) Be specific abut the age ranges for the statistics covered in the introduction. Also, justify why 12-19. The age range is fine, just explain rationale.

2) Mention why school attendance is an outcome as intervention does not seem to target that from current description.

3) Describe why learning disabilities, some of which may be mild, are excluded.

4) Consider audio/video recording and rating a portion of tapes (20%) for intervention fidelity (more of a gold standard than chart reviews)

5) SASH appears to be 6 active sessions. If so, many would not consider that a brief intervention. Also, are sessions weekly (currently unclear)?

6) Why are only certain measures given post-intervention? Would like to read more about the rationale for that choice.

7) 15% attrition accounted for in power calculations. What is that number based upon (previous studies, etc.)? How much attrition do the authors expect at each intervention and assessment visit?

Reviewer **2**

Name **Stenager, Elsebeth**

Affiliation **Psychiatric Research Unit, Aabenraa, Department of Regional Health Services Research, University of Southern Denmark, Denmark; University of Southern Denmark, Odense, Denmark.; Department of Regional Health Research, University of Southern Denmark, Odense, Denmark., Department of regional Health Research**

Date **31-May-2025**

COI **None**

This protocol concerning a randomized controlled trial of a novel brief psychosocial intervention for young people presenting to Emergency departments is in general very well planned and well written, with precise outcome measures. The protocol follow relevant plans for conduction randomized studies.

The background section is precise, leading up to the objectives, however a clear definition of self-harm / suicide attempts would be valuable. Experiences from other ED´s show different assessments of self-harm/ suicide attempts among ED Staff. Clear definitions could help to overcome different assessment in various ED´s. Clear criteria could be included in selection criteria's page 5.

Primary outcome p 9. Line 31-33. It seems a bit confusing that attempted suicide is introduced here as an outcome measure. I think it is important to define self-harm, suicide attempts etc. in the introduction of the study. How is self-harm defined and how is self-harm or suicide attempts with and without intent defined. Maybe a better explanation of the SITBI interview can elucidate this complexity and its solution. As far as I can see, more explanations are found under secondary outcomes. The clinicians in the ED´s should be familiar with these criteria's to avoid big differences in definitions of self-harm.

The protocol follow relevant plans for conduction of randomized studies. The Spirit checklist. The use of the tjecklist should be mentioned under methods and referred to in the text.

Qualitative process evaluation page 15.

The theoretical frame for the assessment of the qualitative study should be explained.

Minor problems:

Page 4, line 37 YP is introduces as a new abbreviation for young people, in the text. Must be introduced previously in the text.

Page 6 line 7,8 explain Tier 3.5.

Page 6, line 41 section about consent under 16 is difficult to understand and should be rewritten.

Table 1. Page 11, content in semi structured interviews and Helping Alliance Questionnaire should be explained better.

VERSION 1 - AUTHOR RESPONSE

Reviewer: 1 Dr. Mary Cwik, Johns Hopkins University

Very clear description of the methods for this important study. A few minor comments for the authors.

Reviewer comments	Response
1) Be specific about the age ranges for the statistics covered in the introduction.	Added to the background section P3:<br><br>"A m**eta-analysis, of which approximately 40% of participants are under 24, found** that presenting to the Emergency Department (ED) with non-fatal self-harm is associated with a nearly 50-fold increased risk of suicide **across all ages** [1]."
Also, justify why 12-19. The age range is fine, just explain rationale.	We have amended the age range to 12-18, as this reflects the ages of young people seen in the services we recruited in. Our age range was initially 12-19, as some CAMHS crisis services in the UK offer care to 19-year-olds. Added to the end of the Introduction section (P5):<br><br>"The age range selected reflects the ages of young people seen by participating services, is in line with other trials of Therapeutic Assessment [2], and takes into account a child's developmental stage in terms of the ability to engage in a psychological intervention."
2) Mention why school attendance is an outcome as intervention does not seem to target that from current description.	Added to p10 in the outcomes section:

	"School attendance is a secondary outcome because of the relationship between self-harm and school absenteeism [26]"
3) Describe why learning disabilities, some of which may be mild, are excluded.	Added to the exclusion criteria (P6): Possible Learning Disability, judged by clinician "due to potential engagement difficulties with consent procedures and study procedures "
4) Consider audio/video recording and rating a portion of tapes (20%) for intervention fidelity (more of a gold standard than chart reviews)	We aimed to rate fidelity using audio/video recordings, however recording was optional and required both practitioner, young person (and carer if present) consent, which was not routinely provided. Some practitioners were reluctant to record sessions before they were confident with the new approach.
5) SASH appears to be 6 active sessions. If so, many would not consider that a brief intervention.<br><br>Also, are sessions weekly (currently unclear)?	This is an interesting point. In the literature there is currently no consensus on the number of sessions which constitute a brief intervention. The SASH intervention is up to 6 sessions. In one of our sites, up to six sessions are delivered as part of standard care (treatment as usual), which is considered a brief crisis intervention.<br><br>P8 includes this sentence: "We aim to deliver the first session within one week of ED attendance. Subsequent sessions are delivered at approximately two-week intervals"<br><br>The session intervals have also been added to the study flow diagram and a new version has been uploaded.
6) Why are only certain measures given post-intervention? Would like to read more about the rationale for that choice.	The Helping Alliance measure is administered once to all participants - after the final session in the intervention arm, and after the 7 day follow-up in the TAU arm. This measure captures the quality of the therapeutic relationship

	with the practitioner. We hypothesise that the quality of the relationship with the practitioner may mediate outcomes.
7) 15% attrition accounted for in power calculations. What is that number based upon (previous studies, etc.)?<br><br><br><br><br><br><br><br><br><br><br><br>How much attrition do the authors expect at each intervention and assessment visit?	The below has been added to P17:<br><br>"Attrition was estimated at 15%, estimated based on 5-25% attrition rate found in similar trials in CYP depending on the duration of follow-up [3,4]."<br><br><br><br><br><br><br><br>The intervention is flexible in terms of the total number of sessions attended by the young person. The young person is considered to have received an adequate therapeutic dose if they receive each of the three elements (Therapeutic Assessment, Enhanced Safety Planning, Solution-Focused practice) which is at least two sessions. If the full six sessions have not been attended, we record whether the young person is adherent, and whether the intervention is complete by mutual agreement or not.<br><br>We expect little attrition at the post-intervention assessment where the Helping Alliance Questionnaire is administered as this is soon after the baseline assessment. We expect around 15% attrition at 6 months follow-up - the rationale for this is provided in the response to comment 7 above.

Reviewer: 2

Dr. Elsebeth Stenager, Psychiatric Research Unit, Aabenraa, Department of Regional Health Services Research, University of Southern Denmark, Denmark; University of Southern Denmark, Odense, Denmark.; Department of Regional Health Research, University of Southern Denmark, Odense, Denmark.

Comments to the Author:

This protocol concerning a randomized controlled trial of a novel brief psychosocial

intervention for young people presenting to Emergency departments is in general very well planned and well written, with precise outcome measures. The protocol follow relevant plans for conduction randomized studies.

Reviewer comments	Response
1. The background section is precise, leading up to the objectives, however a clear definition of self-harm / suicide attempts would be valuable. Experiences from other ED´s show different assessments of self-harm/ suicide attempts among ED Staff. Clear definitions could help to overcome different assessment in various ED´s. Clear criteria could be included in selection criteria's page 5.	Added to the eligibility criteria (P5):<br><br>"presenting in crisis to the ED with self-harm, according to the UK NICE definition, as "intentional self-poisoning or injury irrespective of the apparent purpose of the act" [2], or suicidal ideation with recent self-harm defined as within one month of ED presentation"
2. Primary outcome p 9. Line 31-33. It seems a bit confusing that attempted suicide is introduced here as an outcome measure. I think it is important to define self-harm, suicide attempts etc. in the introduction of the study. How is self-harm defined and how is self-harm or suicide attempts with and without intent defined. Maybe a better explanation of the SITBI interview can elucidate this complexity and its solution. As far as I can see, more explanations are found under secondary outcomes. The clinicians in the ED´s should be familiar with these criteria's to avoid big differences in definitions of self-harm.	The below has been amended on P10:<br><br>"The primary outcome is the number of self-reported episodes of self-harm within the past month, assessed 6 months post-randomisation. This is a count outcome based on summing the number of episodes of self-harm reported from a single item ("How many times in the past month?") across the three behavioural domains (suicide attempt, self-harmed without intent to end life, self-harm with ambiguous intent) from a modified version of the Short Form of the Self Injurious Thoughts and Behaviour Interview (SITBI) [6]. The term 'Suicide Gesture' has been criticized for being dismissive and for potentially minimizing the seriousness of suicidal behaviour. Hence, there is agreement that it should be adapted [7]: we used 'self-harm with ambiguous intent'. Young people are asked to report on the number of times in the past month they have: attempted suicide, self-harmed without intending to end their life or self-harmed where the intent behind the act was ambiguous. These three

	domains encompass all possible episodes of self-harm in line with U.K. NICE definition [5]. The SITBI has been widely used in young people who harm themselves and has strong validity and reliability [6]."
3. The protocol follow relevant plans for conduction of randomized studies. The Spirit checklist. The use of the tjecklist should be mentioned under methods and referred to in the text.	Added to the Methods section in p5:<br><br>"Reporting of methods is in line with the SPIRIT checklist in the Supplementary Material."
4. Qualitative process evaluation page 15.<br>The theoretical frame for the assessment of the qualitative study should be explained.	On page 17 we have specified that we will analysis the qualitative interviews using thematic analysis using the Braun and Clarke method and guidance.
5. Page 4, line 37 YP is introduces as a new abbreviation for young people, in the text. Must be introduced previously in the text.	Corrected
6. Page 6 line 7,8 explain Tier 3.5.	Amended in the exclusion criteria (P6):<br><br>"Need for more intensive treatment than the intervention offers, e.g. **inpatient treatment (tier 4) or intensive or outreach care in the community (**Tier 3.5**)."**
7. Page 6, line 41 section about consent under 16 is difficult to understand and should be rewritten.	Amended for clarity on P7 (previously P6):<br><br>"A guardian with parental responsibility provides consent for young people under the age of 16. Young people participants under the age of 16 indicate their willingness to participate by providing assent after an age-appropriate explanation of the study from a researcher."
8. Table 1. Page 11, content in semi structured interviews and Helping	Amended Table 1 (now on P13), to clarify that the semi-structured interviews with both young people and parent/carers

Alliance Questionnaire should be explained better.	explore their experiences of the intervention:<br><br>“Semi-structured interviews exploring experiences of the intervention”<br><br>Due to limited space in Table 1, a fuller explanation of the Helping Alliance Questionnaire is given on P10:<br><br>Quality of the therapeutic relationship, self-rated by young people participants and measured by the Helping Alliance Questionnaire (HAQ) [8].
